# Etiologies of 46,XY disorders of sex development (DSD): a collaborative study in Hong Kong

**DOI:** 10.1186/1687-9856-2013-S1-P185

**Published:** 2013-10-03

**Authors:** WM But, Angel Chan, CY Lee, Almen Lam, YY Lam, PY Loung, KL Ng, MY Wong, KT Chan, WY Tse, CC Shek

**Affiliations:** 1 Department of Paediatrics, Queen Elizabeth Hospital, Kowloon, Hong Kong; 2 Department of Pathology, Queen Elizabeth Hospital, Kowloon, Hong Kong; 3 Department of Paediatrics, Caritas Medical Centre, Kowloon, Hong Kong; 4 Department of Paediatrics, United Christian Hospital, Kwun Tong, Hong Kong; 5 Department of Paediatrics, Kwong Wah Hospital, Yau Ma Tei, Hong Kong; 6 Department of Paediatrics, Princess Margaret Hospital, Kowloon, Hong Kong

## 

Disorders of sex development (DSD) are defined as congenital conditions in which development of chromosomal, gonadal or anatomical sex is atypical. In 46, XY DSD, the genotype is XY, but the external genitalia is incompletely virilised, ambiguous, or completely female.

The objectives of this prospective study are to evaluate the testicular Sertoli and Leydig cell functions, to establish the genetic basis and to determine the relative prevalence of etiologies in Chinese patients with 46,XY DSD in Hong Kong. All patients with 46,XY DSD (either new or known) presented to five paediatric departments in Hong Kong from July 2009 till June 2011 were recruited. They were assessed by paediatric endocrinologists. Comprehensive evaluation of testicular and adrenal functions was performed using serum hormonal assays and urine steroid profiling. Based on the hormonal results, mutational analyses of the candidate genes by polymerase chain reaction and direct DNA sequencing were conducted to delineate the genetic basis of the etiologies.

Sixty-five patients (54 male and 11 female) with 46,XY DSD were recruited. Their age ranged from birth to 27 years. Sixty-one (94%) patients presented with ambiguous external genitalia, two presented with delayed puberty and one each with primary amenorrhoea and inguinal hernia. Definitive diagnoses were made in 25 (38%) patients. Eleven (17%) patients had 5-alpha reductase 2 deficiency. Androgen insensitivity was confirmed by genetic analysis in eight (12%) patients. There was one patient with each of the following etiologies: Swyer syndrome, SF-1 mutation, Frasier syndrome, cholesterol side-chain cleavage deficiency, persistent Mullerian duct syndrome and mixed gonadal dysgenesis. Genetic basis of the etiologies was delineated in 23 (35%) patients. A total of 10 novel mutations were identified. The longest follow up period was 27 years, none of the patients requested change of gender sex so far. In conclusion, 46,XY DSD is a heterogeneous group with diverse etiologies. Although 5-alpha reductase 2 deficiency is believed to be rare, it is not uncommon in Hong Kong.

